# Olympic Games Event Recognition via Transfer Learning with Photobombing Guided Data Augmentation

**DOI:** 10.3390/jimaging7020012

**Published:** 2021-01-20

**Authors:** Yousef I. Mohamad, Samah S. Baraheem, Tam V. Nguyen

**Affiliations:** 1 Department of Computer Science, University of Dayton, Dayton, OH 45469, USA; baraheems1@udayton.edu (S.S.B.); tamnguyen@udayton.edu (T.V.N.); 2 Department of Computer Science, College of Computer Technology Benghazi, Benghazi 16063, Libya

**Keywords:** image classification, sport event classification, transfer learning, deep learning, data augmentation

## Abstract

Automatic event recognition in sports photos is both an interesting and valuable research topic in the field of computer vision and deep learning. With the rapid increase and the explosive spread of data, which is being captured momentarily, the need for fast and precise access to the right information has become a challenging task with considerable importance for multiple practical applications, i.e., sports image and video search, sport data analysis, healthcare monitoring applications, monitoring and surveillance systems for indoor and outdoor activities, and video captioning. In this paper, we evaluate different deep learning models in recognizing and interpreting the sport events in the Olympic Games. To this end, we collect a dataset dubbed Olympic Games Event Image Dataset (OGED) including 10 different sport events scheduled for the Olympic Games Tokyo 2020. Then, the transfer learning is applied on three popular deep convolutional neural network architectures, namely, AlexNet, VGG-16 and ResNet-50 along with various data augmentation methods. Extensive experiments show that ResNet-50 with the proposed photobombing guided data augmentation achieves 90% in terms of accuracy.

## 1. Introduction

Sports are a major section of media, accounting for a massive portion of TV broadcasting, and they have become a dominant focus in the field of entertainment, thanks to the massive commercial appeal of sports programs [1]. With this rapid increase and the explosive spread of sport data, the need for fast and accurate access to the right information has become a challenging task with considerable importance for multiple practical applications. Sport activities that are captured by computer vision and deep learning applications can be used for tactical analysis, athlete detection, movement analysis, pose estimation, and tracking [2]. Therefore, research in event recognition has witnessed a large and rapid increase.

**Figure 1:**
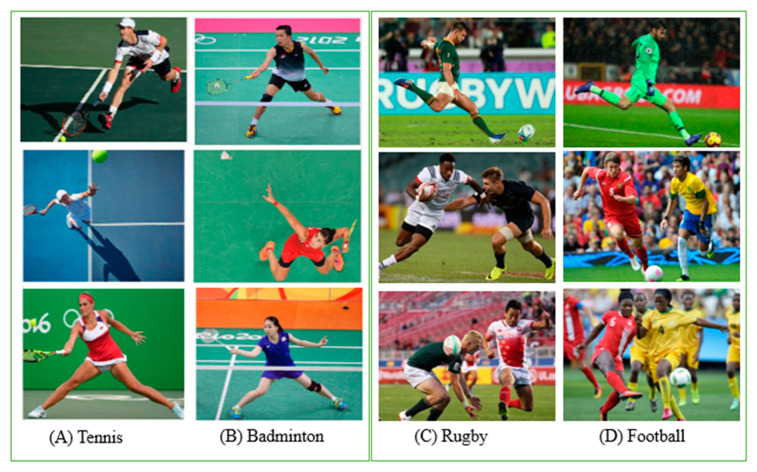
OGED dataset images. Illustration of similarities between Tennis and Badminton (**A**,**B**) and between Football and Rugby (**C**,**D**), which can be easily misclassified.

Convolutional Neural Networks (CNNs) have shown advances in the field of image recognition. In addition to the improvement in image classification, CNNs have made magnificent progress in object detection and key point prediction [3]. However, sport event recognition at the Olympic Games is still a challenging problem due to the pattern similarities in many sports. These similarities include outfit, equipment, and game field. For example, as shown in Figure 1, Tennis and Badminton share numerous features, including racquets, field outline and color, net, and outfit, and so do Football and Rugby. Such scenarios are confusing for most traditional CNNs, which makes it a challenging task for convolutional networks to classify these types of sport events successfully and correctly. 

To this end, we first collect an Olympic Games Event Image Dataset (OGED), including 10 different sport events scheduled for the Olympic Games Tokyo 2020 (Tokyo 2020 Olympics was officially postponed until 2021 due to the coronavirus pandemic). Specifically, the OGED dataset consists of 1000 images of Athletics, Badminton, Basketball, Football, Handball, Rugby, Swimming, Tennis, Water polo, and Weightlifting. Then, we conduct transfer learning on three popular deep learning models, namely, AlexNet [4], VGG-16 [5], and ResNet-50 [6]. AlexNet was named after the main author—**Alex** Krizhevsky. Meanwhile VGGNet is named after the **V**isual **G**eometry **G**roup at Oxford University. ResNet is the short name of **Res**idual **Net**work. Transfer learning is the process of reusing the pre-trained model that is trained on a very huge dataset as a starting point for training another small dataset, where the weights are reused as initial weights. Thus, the training time is greatly reduced, and the accuracy is greatly increased. Therefore, we use the transfer learning technique to train OGED to recognize the Olympic Games. In addition, we propose photobombing guided data augmentation, which consists of geometric data augmentation and random erasing augmentation techniques, in order to improve the recognition accuracy. We use the geometric data augmentation technique to perform some transformations on the dataset such as rotation, translation, shearing, and horizontal flipping. The reason for this is to increase the size of our OGED dataset with different transformation aspects in order to boost the performance. Random erasing augmentation is the process of selecting a random rectangle region in an image and replacing it with random pixel values to overcome the overfitting problem and increase the accuracy. Last but not least, we conduct extensive experiments to evaluate the performance of different backbones and data augmentation methods on OGED dataset. 

## 2. Related Work

Sport analysis has increasingly been a research point of interest in the last two decades. In the survey conducted by Shih [1], it is indicated that surveys and published work in this field have experienced rapid growth, where these surveys were conducted to inspect the results of different research topics. Some of these topics include computer-assisted referral, highlight extraction, tactic summarization, and content insertion. Initially, image classification started with implementing hand-crafted features followed by the traditional machine learning pipeline. However, the performance and results were not promising when they were applied on large datasets. Therefore, scientist and researchers have developed various deep learning models and methods to efficiently classify huge datasets based on different input types including images and videos. The breakthrough in training unsupervised neural networks was when Hinton et al. [7] introduced Deep Belief Networks (DBNs) in 2006. Myriad methods have been developed by the researchers to enhance CNN architectures. One of the most prominent methods is AlexNet, which was proposed by Alex Krizhevsky et al. in 2012 [4]. This state-of-the-art method was developed and successfully implemented in ImageNet Challenge to classify large-scale images. Since then, other deep learning models such as GoogLeNet, ResNet, and VGGNet have been widely adopted in both image and video analysis [5,8,9,10,11]. With the immense advancement of convolutional neural networks, there have been various methods developed to classify sport events using either spatial features or temporal features as inputs. However, a rather small number of those methods have been used in the classification of various game events such as the Olympic Games. Most of the previously proposed methods focused on a single game or several non-Olympic games, such as the authors in [9]; with their attempt to classify sport videos, they formulated their general model using combined method to solve classification problem of sport dataset where they implemented Convolutional Neural Network (CNN) to extract the features of the dataset and then combined these features with temporal information from Recurrent Neural Network (RNN). The highest achieved rate was 94% after applying transfer learning with the VGG-16 model. Similarly, the CNN classification model, along with deep transfer learning, was used on a single type of sport Video Scene, that is, the Soccer and Event Dataset, in [12], but an accuracy of only 89% was achieved. Finally, the method in [13] conducted ball-by-ball outcome classification for cricket sport videos, where there were four different classification predictions for each ball based on the outcome of video frame sequences of the ball action. A Long-Term Recurrent Convolutional Network using a pretrained VGG16Net, the LRCN architecture, and a single frame-based architecture, are three different Convolutional Neural Network architectures, the performance of which was compared against each other. In our research work, we have applied extensive experiments using transfer learning and fine-tuning the pre-trained weights of AlexNet [4], VGG-16 [5], and ResNet-50 [6]. We chose AlexNet, VGG-16, and ResNet-50 due to their promising results on ImageNet Large-Scale Visual Recognition Challenge (ILSVRC) [14], where these state-of-the-art image classifiers scored in the top five with respect to accuracy on the ImageNet dataset. In addition, the three chosen models are the most common learning models recommended in [15,16] for various computer vision tasks. A quick overview of these three models is discussed below.

AlexNet as one of the milestones, and was proposed by Krizhevsky et al. [4] in 2012. It was successfully applied to large-scale image classification in the well-known ImageNet Challenge. AlexNet was employed as a source architecture for solving the human action recognition problem by Sargano et al. [8]. Please note that the human action recognition conducted in [8] relied only on the spatial information. Specifically, the ImageNet dataset was used to train AlexNet, where 224 × 224 pixels of a colored image are taken as an input and classified into the corresponding class. Five convolutional layers, C1–C5, form the architecture of AlexNet, followed by three fully connected layers FC6–FC8. This Deep Convolutional Neural Networks used in [8] is one of the networks that we use in our work where fine-tuning method is used for optimizing the recognition task result.

VGG-16 network consists of 16 convolution layers with the size of 3 × 3 convolution kernels that are stacked on top of each other, resulting in an increase in the depth of the network. VGG-16 uses Max Pooling layers to reduce the volume size where the size of Max Pooling kernels is 2 × 2. The last two layers in VGG-16 are fully connected layers of 4096 neurons each, and then the last layer is a softmax classifier to obtain the respected class of the input.

ResNet-50 is a ResNet variant [6] with 50 layers and is used most frequently in transfer learning. While ResNet has a similar architecture of VGG-16, it uses additional identity mapping that allows the model to bypass a convolutional network weight layer in case the current layer does not substantially reduce the overfitting problem. Moreover, ResNet uses shortcut path alternation to reduce the problem of vanishing gradients [6], due to which it was extremely difficult to train very deep neural networks prior to ResNet [17].

Therefore, the objective from using and fine-tuning these three models with multiple augmentation methods in our research is to experiment the highest validation accuracy for classification of spatial features of the OGED dataset. We use different augmentation techniques on these three models to enhance and boost the accuracy to the highest rate.

The remainder of this paper is organized as follows: Section 3 introduces data collection and the proposed system, while Section 4 introduces the experimental settings and evaluation process. Finally, Section 5 draws the conclusions and paves the way for future work.

## 3. Data Collection and Proposed Framework

### 3.1. Data Collection: Olympic Games Event Dataset (OGED)

Due to the lack of availability of a specific Olympic Games dataset, the novel OGED generated in this project is an essential step. This dataset is used for training and testing purposes. This dataset will be made public along with the publication.

The OGED dataset consists of 1000 labeled images belonging to 10 different Olympic Games events: Athletics, Badminton, Basketball, Football, Handball, Rugby, Swimming, Tennis, Water polo, and Weightlifting. Please note that these sports events are officially scheduled for Tokyo 2020 [18]. All images have the same resolution of 1366 × 768 pixels based on the frame captured from the Olympic Channel on YouTube, where the rate of these frames was 25 fps. The OGED dataset is annotated, balanced, and is distributed evenly with 100 images per class and a total of 1000 images for the entire dataset. 80% of the OGED images were used for training the networks and 20% were used for testing. The images in this dataset were collected and randomly captured from YouTube videos of real Olympic Games featuring different events, ranging from Atlanta 1996 to the most recent in Rio 2016 due to the availability of video data. The OGED dataset collection represents a natural pool of actions featured in a wide range of scenes and viewpoints of the games and depicts the Olympic events under many viewing conditions. These conditions include the position of the athletes, camera viewpoints, and variation of the scale. Furthermore, the dataset is subjected to several augmentation methods, including the geometric augmentation of performing rotation, translation, shearing, and flipping the images to increase the size of the dataset and to minimize overfitting of the training model. Moreover, we apply the photobombing guided data augmentation on our OGED dataset to increase the dataset further and hence enhance the performance. Details about the data augmentation techniques are presented in the next subsection.

### 3.2. Photobombing Guided Data Augmentation

Since our OGED dataset contains 1000 images of different Olympic Games events, data augmentation needs to be applied to overcome the overfitting problem. We first start the model transfer learning without any data augmentation. Then, we apply simple geometric augmentation to our OGED by performing rotation, translation, shearing, and horizontal flipping to improve the performance. We also apply Random Erasing [19] as a data augmentation method. In particular, Random Erasing selects a random rectangle region in an image and replaces it with random pixel values. 

**Figure 2:**
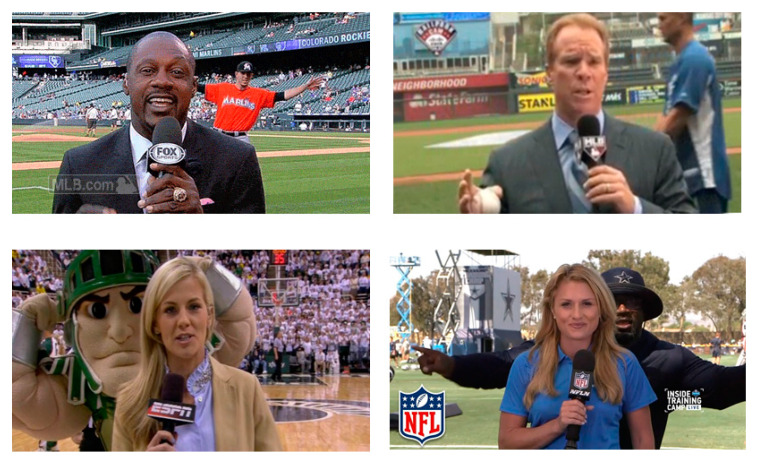
Examples of photobombing in real sport events.

**Figure 3:**
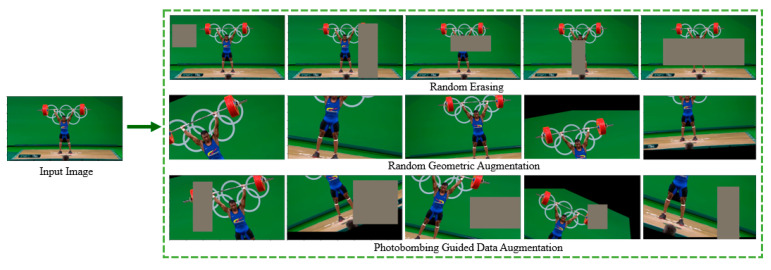
Examples of data augmentation methods used in this research including photobombing guided data augmentation.

Actually, photobombing takes place very often in sport photos. According to the Oxford dictionary [20], photobombing is the act of purposely putting oneself into the view of a photograph, often in order to play a practical joke on the photographer or the subjects. Figure 2 shows examples of photobombing in real sport events. To simulate photobombing, we combine the random geometric augmentation with random erasing augmentation. Figure 3 illustrates different data augmentations examined in this paper, namely, geometric augmentation, random erasing augmentation and the proposed photobombing guided data augmentation.

### 3.3. Transfer Learning Using Pre-Trained Models

**Figure 4:**
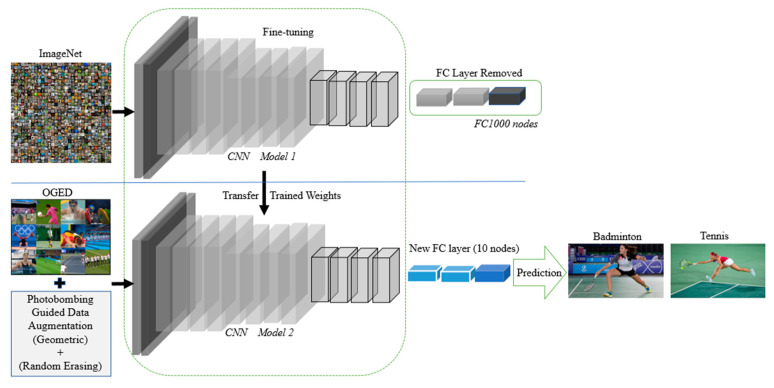
The pipeline of our proposed framework for event recognition via transfer learning and photobombing guided data augmentation.

In real-world applications, training a new deep learning model from scratch is very time consuming, costly, and requires high computational resources, a huge amount of data, and hours or perhaps days of training [21]. Therefore, to solve this issue, transfer learning is an appropriate solution. Transfer learning falls under two categories: transfer learning using feature extraction and transfer learning using fine-tuning methods [22]. The second type of transfer learning is the method that we use in this work, since it has been proven that it can outperform the feature extraction method. Then, in this case, we have to decide between the massive datasets such as ImageNet (ILSVRC) [14] or MS-COCO dataset [23] for our model to be pre-trained on. The chosen pre-trained weights for our framework is the ImageNet dataset, where we use VGG-16 [5], ResNet-50 [6], and AlexNet [4] models, respectively, pre-trained on ImageNet. The input image size of VGG-16 is 224 × 224, and the same for ResNet-50, while it is 227 × 227 for AlexNet. Figure 4 visualizes the architecture of fine-tuning AlexNet, VGG-16, and ResNet-50 on our OGED dataset.

With respect to fine-tune AlexNet [4], we again apply transfer learning [4]. The structure goes through two steps. In the first step, the parameters in the pre-trained AlexNet are marginally tweaked, so that they are adapted to the OGED images. The second step is fine-tuning AlexNet, where the fully connected layer in AlexNet is replaced with a new fully connected layer that uses a softmax classifier with 10 classes of our OGED. The softmax activation function is used for the multi-class classification problem, and it is a general binary form of Logistic Regression. This is like hinge loss or squared hinge loss that uses mapping function *f* that maps the input *x*, which could be the image pixels or the extracted features, to the class label output *y* via a linear operation of dot product of the input *x* and corresponding weight matrix *W*, as shown in Equation (1).

(1) $$f\left(x_{i},W\right)=x_{i}*W$$

However, it is different from hinge loss, where for each class label, the score is calculated via unnormalized log probabilities in order to switch to cross-entropy loss function instead of hinge loss function as illustrated in Equation (2).

(2) $$L_{i\;}=\;-\log\left(e^{s_{y_{i}}}\;/\;\sum^{\text{​}}j^{e^{s_{j}}}\right)$$

where $\;s=f(x_{i}$, *W*). Please note that *W* is updated via our transfer learning with new data.

Moreover, we adapt a small learning rate, so the weights of our network are not dramatically changed. At the same time, the fully connected layer weights are randomly initialized. Furthermore, the weights of the CNN are updated using the Stochastic Gradient Descent (SGD) algorithm based on the OGED dataset. We train the model on OGED for 25 epochs. Finally, we apply all three methods of augmentation on the OGED dataset to increase the total number of images during the training process, and we evaluate the performance of the three different augmentation methods.

Similarly, in order to fine-tune VGG-16, VGG-16 [5] with weights pre-trained on ImageNet are loaded, and the fully connected FC layer head is removed. This FC layer is replaced with a newly created FC layer that contains 10 neurons for 10 classes of our OGED dataset and uses softmax as the activation function. Then, earlier CONV layers in the network are frozen to preserve the features learned previously by the convolutional neural network to allow the gradient to backpropagate through the FC layers. Now, the training process on the OGED dataset is initiated for 50 epochs from the freshly added FC layer head where the weights are updated. Finally, when the OGED dataset patterns are being learned by the fully connected layer, the last set of the CONV layers is unfrozen, and the whole model is retrained for 20 epochs to include the last CONV layers and the fully connected layer. Then, we continue evaluating our model where the learning rate is slightly progressing until an optimum accuracy is obtained. We run the model again three more times while applying geometric data augmentation, randomly erasing data augmentation, and the combination of both augmentation methods on our OGED dataset and record the results.

Regarding fine-tuning ResNet50 [6], we use the same concept of transfer learning that is used with VGG-16 [5]. We start by loading ResNet-50 [6] and the weights that were pre-trained on ImageNet. We remove the fully connected layer head from the original architecture as well. From there, new head layers are constructed and appended on top of the ResNet-50 layers, where average pooling and fully connected layer with our OGED class labels and softmax activation function are applied. Next, we freeze the weights of the base neural network model and allow only the network head to be trainable for 50 epochs prior to unfreezing the last set of CONV layers and retraining the model for 20 epochs. Eventually, we evaluate the model on OGED testing data to make predictions. Furthermore, we rerun the model again three more times, as we did with the VGG-16 [5] model in order to apply geometric data augmentation, randomly erasing data augmentation, and the combination of both methods of augmentation on our OGED dataset and evaluate the accuracy results.

## 4. Experimental Results

### 4.1. Experimental Settings

In this section, we conduct several experiments on our collected dataset (OGED). We first evaluate the performance of pre-trained models on the ImageNet dataset [18], VGG-16 [5], ResNet-50 [6], and AlexNet [4], using K-Nearest Neighbor (KNN). We then fine-tune these pre-trained models with OGED. In particular, we consider several fine-tuning variants during the experiments as follows.

- Fine-tuning the network without data augmentation.
- Fine-tuning the network with geometric data augmentation technique where we perform rotation, translation, shearing, and horizontal flipping.
- Fine-tuning the network with randomly erased data augmentation technique [19].
- Fine-tuning the network with the proposed photobombing guided data augmentation.

The accuracy is used as the main metric to measure the performance.

### 4.2. Performance of Pre-Trained Models with KNN

In this step, we conduct an experiment to see how good the learned features of the pre-trained models—VGG-16, ResNet50, and AlexNet—using extracted features and training them using K-Nearest Neighbor (KNN). We start by extracting the features from the last three layers of each of three finetuned models. Then, we apply the KNN classifier on top of each of these three models and use it for predicting the classes of our OGED test set. In the KNN classifier, we use various values of K, specifically, 1, 3, 5, and 10, to experiment with different Ks on the extracted features. As can be seen in Table 1, the extracted features perform efficiently on the KNN classifier, and as expected, ResNet-50 achieved the highest performance, with 73% accuracy. Meanwhile, VGG-16 had the second highest accuracy, with 68%, followed by AlexNet, with only 55% accuracy rate using KNN.

### 4.3. Experimental Results on OGED

**Figure 5:**
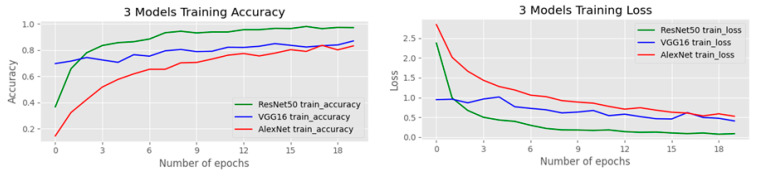
Comparison of the training accuracy and training loss on the OGED dataset.

In this subsection, we conduct experiments with our transfer learning on the three deep learning models. We fine-tune AlexNet [4], VGG-16 [5], and ResNet-50 [6] leveraging different techniques during the fine-tuning process. Figure 5 shows the comparison plots of the three models for training accuracy and training loss on the OGED dataset. The accuracy results of our experiments are illustrated in Table 1. The highest accuracy in each network is achieved when we combine both data augmentation and randomly erased data augmentation [19] methods. Furthermore, the best accuracy among all settings in all three networks is achieved when fine-tuning ResNet-50 [6] using photobombing guided data augmentation, resulting in 90% accuracy. 

**Figure 6:**
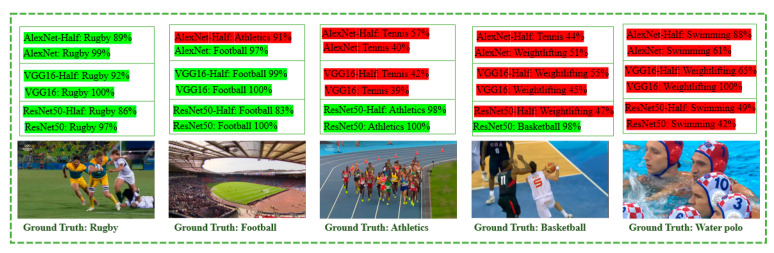
Classification visualization examples of AlexNet [4], VGG-16 [5], and ResNet50 [6] models on OGED test set. The correct and incorrect classification results are marked in green and red, respectively.

With respect to the failure cases of the three models, Figure 6 shows the failure classification results across all of the three models and in different class labels. For example, as can be seen in the case of AlexNet, it misclassifies Basketball with Handball, and Rugby with Football. Furthermore, we note with ResNet50 that there are undesired classification results, where Water polo is misclassified as Swimming and Athletics is confused with Weightlifting. Finally, in the case of VGG-16, Basketball is misclassified as Rugby, and Tennis in this particular image shot is confused with Weightlifting. The reason for these misclassification results is that the three networks misclassify these specific image scenes due to their large pattern similarities. Regardless of the fact that some classes have an individual low performance when it comes to accuracy rate, the overall performance is promising, and the accuracy percentage is high and satisfying. 

## 5. Conclusions and Future Work

In this work, we introduce a framework for Olympic Games event recognition via transfer learning and photobombing guided data augmentation. To this end, we first collect an Olympic Games Event image Dataset (OGED) that contains 1000 images categorized into 10 different sport events. The OGED dataset is collected and randomly captured from YouTube videos of real Olympic Games. We then employ pre-trained convolutional neural networks, specifically, AlexNet, VGG-16, and ResNet-50, with photobombing guided data augmentation on our newly collected dataset OGED. To validate the capabilities of our proposed methods and architectures, the OGEG undergoes three different data augmentation methods. First, we fine-tune each model without any data augmentation methods. Then, we fine-tune the network using either geometric data augmentation or random erasing data augmentation, and lastly, we train the model after applying the photobombing guided data augmentation method, which is the combination of both augmentation techniques. The experimental results show that the highest accuracy is achieved in all architectures with photobombing guided data augmentation with 84%, 85%, and 90% for AlexNet, VGG-16, and ResNet-50 respectively. Among different backbones, ResNet-50 achieves the highest accuracy in all of the experimental results with 90% accuracy. 

In the future, we are interested in investigating more data augmentation methods [24], and implementing more state-of-the-art networks for transfer learning. It is worth noting that the learned models aim to map the data manifold *X*, i.e., training data, onto another manifold *Y*, i.e., training labels. If there exists noise in the training data, the performance of the learned models will be affected. Thus, we would like to investigate the impact of data contamination and data augmentation in training deep learning models. In addition, the learned models are lacking in explainability. Therefore, we are interested in incorporating object detectors (human detector, sport equipment detector), human pose estimation, and scene segmentation to better understand and explain the recognized sport events.

## Figures and Tables

**Table 1:** Experimental results of different backbones and data augmentation methods. The best performance of each backbone is marked with boldface.

Augmentation Method	AlexNet [4]	VGG-16 [5]	ResNet-50 [6]
Pre-trained Model—KNN (N = 1)	48%	56%	71%
Pre-trained Model—KNN (N = 3)	54%	60%	66%
Pre-trained Model—KNN (N = 5)	53%	62%	72%
Pre-trained Model—KNN (N = 10)	50%	66%	73%
Transfer Learning—without any data augmentation	73%	79%	82%
Transfer Learning—with geometric data augmentation	83%	84%	87%
Transfer Learning—with randomly erased data augmentation [19]	76%	82%	89%
Transfer Learning—with photobombing guided data augmentation	**84%**	**85%**	**90%**
